# Supplementary material for: Genomic analysis of Candida auris transmission within an urban region
Source: Microb Genom. 2025 Sep 10;11(9):001478. doi: 10.1099/mgen.0.001478 (PMC12452193; doi:10.1099/mgen.0.001478)
Supplement: Uncited Fig. S1. [file mgen-11-01478-s001.pdf]

SUPPLEMENTARY INFORMATION

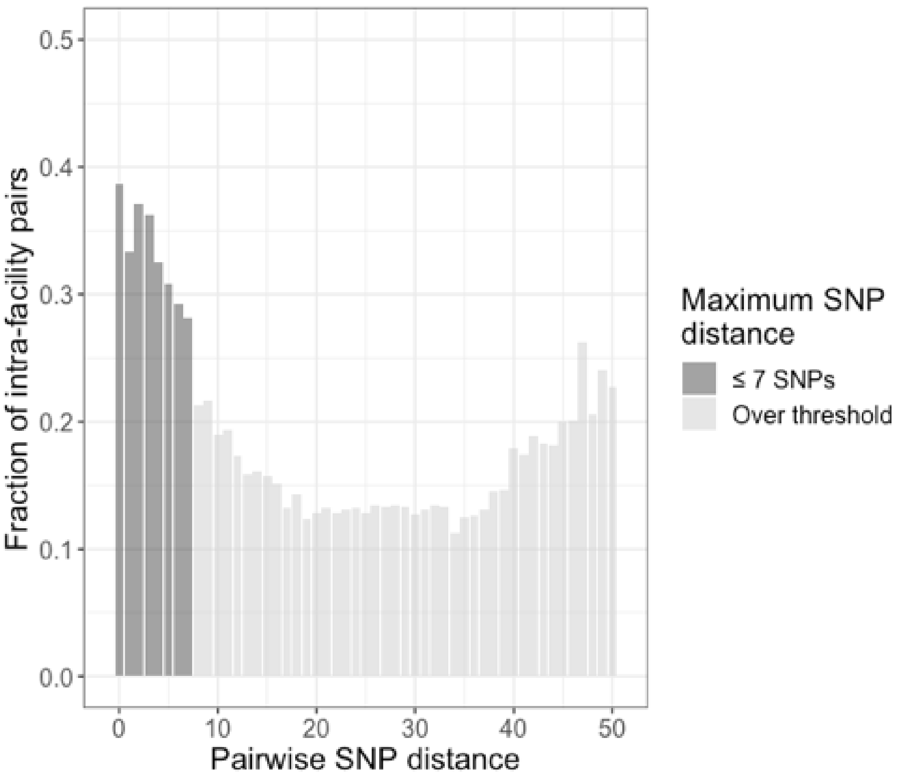

Fig. S1. Maximum SNP distance selection based on SNP distance of intra-facility pairs. The fraction of pairwise comparisons that are intra-facility at each pairwise SNP distance. Selected maximum SNP distance for cluster detection is shaded.

Table S1. *Candida auris* sequence data included in genomic analysis

Sequence ID	SRA Accession	Sequence Type	C. auris clade
B11205	SRR3883436	Clade reference	I
B11220	SRR3883452	Clade reference	II
B11221	SRR3883453	Clade reference	III
B11243	SRR3883464	Clade reference	IV
B11842	SRR7909141	Illinois contextual	IV
B11843	SRR7909220	Illinois contextual	IV
B11880	SRR7909238	Illinois contextual	IV
B11882	SRR7909309	Illinois contextual	IV

B11883	SRR7909391	Illinois contextual	IV
B11884	SRR7909250	Illinois contextual	IV
B11885	SRR7909191	Illinois contextual	IV
B11886	SRR7909407	Illinois contextual	IV
B12028	SRR7909256	Illinois contextual	IV
B12029	SRR7909248	Illinois contextual	IV
B12030	SRR7909228	Illinois contextual	IV
B12031	SRR7909172	Illinois contextual	IV
B12032	SRR7909387	Illinois contextual	IV
B12033	SRR7909179	Illinois contextual	IV
B12034	SRR7909388	Illinois contextual	IV
B12035	SRR7909335	Illinois contextual	IV
B12036	SRR7909151	Illinois contextual	IV
B12046	SRR7909366	Illinois contextual	IV
B12077	SRR7909138	Illinois contextual	IV
B12189	SRR7909158	Illinois contextual	IV
B12190	SRR7909245	Illinois contextual	IV
B12229	SRR7909200	Illinois contextual	IV
B12378	SRR7909145	Illinois contextual	IV
B12380	SRR7909294	Illinois contextual	IV
B12388	SRR7909221	Illinois contextual	IV
B12406	SRR7909405	Illinois contextual	IV
B12510	SRR7909227	Illinois contextual	IV
B12579	SRR7909315	Illinois contextual	IV
B12937	SRR7909226	Illinois contextual	IV
B12938	SRR7909357	Illinois contextual	IV
B20665	SRR23919790	Illinois contextual	IV
B20666	SRR23919789	Illinois contextual	IV
B21737	SRR23919786	Illinois contextual	IV
B21738	SRR23919785	Illinois contextual	IV
B21739	SRR23919784	Illinois contextual	IV
B21740	SRR23919783	Illinois contextual	IV
B21741	SRR23919782	Illinois contextual	IV
B21742	SRR23919781	Illinois contextual	IV
B21743	SRR23919780	Illinois contextual	IV
B21744	SRR23919779	Illinois contextual	IV
B21745	SRR23919788	Illinois contextual	IV
B21746	SRR23919787	Illinois contextual	IV
CA01	SRR12363130	Illinois contextual	IV
CA02	SRR12363129	Illinois contextual	IV

CA03	SRR12363163	Illinois contextual	IV
CA04	SRR12363162	Illinois contextual	IV
CA05	SRR12363161	Illinois contextual	IV
CA06	SRR12363160	Illinois contextual	IV
CA07	SRR12363159	Illinois contextual	IV
CA08	SRR12363158	Illinois contextual	IV
CA09	SRR12363157	Illinois contextual	IV
CA10	SRR12363156	Illinois contextual	IV
CA11	SRR12363155	Illinois contextual	IV
CA12	SRR12363154	Illinois contextual	IV
CA13	SRR12363152	Illinois contextual	IV
CA14	SRR12363151	Illinois contextual	IV
CA15	SRR12363150	Illinois contextual	IV
CA16	SRR12363149	Illinois contextual	IV
CA17	SRR12363148	Illinois contextual	IV
CA18	SRR12363147	Illinois contextual	IV
CA19	SRR12363146	Illinois contextual	IV
CA20	SRR12363145	Illinois contextual	IV
CA21	SRR12363144	Illinois contextual	IV
CA22	SRR12363143	Illinois contextual	IV
CA23	SRR12363141	Illinois contextual	IV
CA24	SRR12363140	Illinois contextual	IV
CA25	SRR12363139	Illinois contextual	IV
IL-RIPHL_CAU-017070-0401	SRR29662561	New sequence	IV
IL-RIPHL_CAU-018070-0404	SRR29662496	New sequence	IV
IL-RIPHL_CAU-018070-0405	SRR29662485	New sequence	IV
IL-RIPHL_CAU-018070-0406	SRR29662474	New sequence	IV
IL-RIPHL_CAU-018070-0407	SRR29662463	New sequence	IV
IL-RIPHL_CAU-018070-0408	SRR29662452	New sequence	IV
IL-RIPHL_CAU-018070-0409	SRR29662441	New sequence	IV
IL-RIPHL_CAU-018070-0450	SRR29662384	New sequence	IV
IL-RIPHL_CAU-018070-0462	SRR29662372	New sequence	IV
IL-RIPHL_CAU-030070-0402	SRR29662550	New sequence	IV
IL-RIPHL_CAU-030070-0457	SRR29662377	New sequence	IV
IL-RIPHL_CAU-030070-0458	SRR29662376	New sequence	IV
IL-RIPHL_CAU-030070-0459	SRR29662375	New sequence	IV
IL-RIPHL_CAU-033070-0391	SRR29662573	New sequence	IV
IL-RIPHL_CAU-033070-0392	SRR29662430	New sequence	IV
IL-RIPHL_CAU-033070-0403	SRR29662507	New sequence	IV
IL-RIPHL_CAU-033070-0502	SRR29662562	New sequence	IV

IL-RIPHL_CAU-033070-0503	SRR29662560	New sequence	IV
IL-RIPHL_CAU-033070-0504	SRR29662559	New sequence	IV
IL-RIPHL_CAU-033070-0505	SRR29662558	New sequence	IV
IL-RIPHL_CAU-033070-0506	SRR29662557	New sequence	IV
IL-RIPHL_CAU-033070-0507	SRR29662556	New sequence	IV
IL-RIPHL_CAU-033070-0508	SRR29662555	New sequence	IV
IL-RIPHL_CAU-033070-0509	SRR29662554	New sequence	IV
IL-RIPHL_CAU-033070-0510	SRR29662553	New sequence	IV
IL-RIPHL_CAU-033070-0511	SRR29662552	New sequence	IV
IL-RIPHL_CAU-034070-0559	SRR29662469	New sequence	IV
IL-RIPHL_CAU-035070-0390	SRR29662574	New sequence	IV
IL-RIPHL_CAU-035070-0537	SRR29662491	New sequence	IV
IL-RIPHL_CAU-035070-0538	SRR29662490	New sequence	IV
IL-RIPHL_CAU-035070-0539	SRR29662489	New sequence	IV
IL-RIPHL_CAU-035070-0540	SRR29662488	New sequence	IV
IL-RIPHL_CAU-035070-0541	SRR29662487	New sequence	IV
IL-RIPHL_CAU-035070-0542	SRR29662486	New sequence	IV
IL-RIPHL_CAU-035070-0543	SRR29662484	New sequence	IV
IL-RIPHL_CAU-035070-0544	SRR29662483	New sequence	IV
IL-RIPHL_CAU-035070-0545	SRR29662482	New sequence	IV
IL-RIPHL_CAU-035070-0546	SRR29662481	New sequence	IV
IL-RIPHL_CAU-035070-0547	SRR29662480	New sequence	IV
IL-RIPHL_CAU-035070-0548	SRR29662479	New sequence	IV
IL-RIPHL_CAU-035070-0549	SRR29662478	New sequence	IV
IL-RIPHL_CAU-035070-0550	SRR29662477	New sequence	IV
IL-RIPHL_CAU-035070-0551	SRR29662476	New sequence	IV
IL-RIPHL_CAU-035070-0552	SRR29662475	New sequence	IV
IL-RIPHL_CAU-035070-0553	SRR29662473	New sequence	IV
IL-RIPHL_CAU-035070-0554	SRR29662472	New sequence	IV
IL-RIPHL_CAU-035070-0555	SRR29662471	New sequence	IV
IL-RIPHL_CAU-036070-0529	SRR29662500	New sequence	IV
IL-RIPHL_CAU-036070-0530	SRR29662499	New sequence	IV
IL-RIPHL_CAU-036070-0531	SRR29662498	New sequence	IV
IL-RIPHL_CAU-036070-0532	SRR29662497	New sequence	IV
IL-RIPHL_CAU-036070-0533	SRR29662495	New sequence	IV
IL-RIPHL_CAU-036070-0534	SRR29662494	New sequence	IV
IL-RIPHL_CAU-036070-0535	SRR29662493	New sequence	IV
IL-RIPHL_CAU-036070-0536	SRR29662492	New sequence	IV
IL-RIPHL_CAU-037070-0397	SRR29662371	New sequence	IV
IL-RIPHL_CAU-037070-0398	SRR29662360	New sequence	IV

IL-RIPHL_CAU-037070-0399	SRR29662349	New sequence	IV
IL-RIPHL_CAU-037070-0400	SRR29662572	New sequence	IV
IL-RIPHL_CAU-037070-0410	SRR29662429	New sequence	IV
IL-RIPHL_CAU-037070-0412	SRR29662418	New sequence	IV
IL-RIPHL_CAU-037070-0414	SRR29662535	New sequence	IV
IL-RIPHL_CAU-037070-0415	SRR29662524	New sequence	IV
IL-RIPHL_CAU-037070-0416	SRR29662517	New sequence	IV
IL-RIPHL_CAU-037070-0417	SRR29662516	New sequence	IV
IL-RIPHL_CAU-037070-0418	SRR29662515	New sequence	IV
IL-RIPHL_CAU-037070-0419	SRR29662514	New sequence	IV
IL-RIPHL_CAU-037070-0420	SRR29662513	New sequence	IV
IL-RIPHL_CAU-037070-0421	SRR29662512	New sequence	IV
IL-RIPHL_CAU-037070-0422	SRR29662414	New sequence	IV
IL-RIPHL_CAU-037070-0423	SRR29662413	New sequence	IV
IL-RIPHL_CAU-037070-0424	SRR29662412	New sequence	IV
IL-RIPHL_CAU-037070-0425	SRR29662411	New sequence	IV
IL-RIPHL_CAU-037070-0426	SRR29662410	New sequence	IV
IL-RIPHL_CAU-037070-0427	SRR29662409	New sequence	IV
IL-RIPHL_CAU-037070-0428	SRR29662408	New sequence	IV
IL-RIPHL_CAU-037070-0429	SRR29662407	New sequence	IV
IL-RIPHL_CAU-037070-0430	SRR29662406	New sequence	IV
IL-RIPHL_CAU-037070-0431	SRR29662405	New sequence	IV
IL-RIPHL_CAU-037070-0432	SRR29662403	New sequence	IV
IL-RIPHL_CAU-037070-0433	SRR29662402	New sequence	IV
IL-RIPHL_CAU-037070-0434	SRR29662401	New sequence	IV
IL-RIPHL_CAU-037070-0435	SRR29662400	New sequence	IV
IL-RIPHL_CAU-037070-0436	SRR29662399	New sequence	IV
IL-RIPHL_CAU-037070-0437	SRR29662398	New sequence	IV
IL-RIPHL_CAU-037070-0438	SRR29662397	New sequence	IV
IL-RIPHL_CAU-037070-0439	SRR29662396	New sequence	IV
IL-RIPHL_CAU-037070-0440	SRR29662395	New sequence	IV
IL-RIPHL_CAU-037070-0441	SRR29662394	New sequence	IV
IL-RIPHL_CAU-037070-0442	SRR29662392	New sequence	IV
IL-RIPHL_CAU-037070-0443	SRR29662391	New sequence	IV
IL-RIPHL_CAU-037070-0444	SRR29662390	New sequence	IV
IL-RIPHL_CAU-037070-0445	SRR29662389	New sequence	IV
IL-RIPHL_CAU-037070-0446	SRR29662388	New sequence	IV
IL-RIPHL_CAU-037070-0447	SRR29662387	New sequence	IV
IL-RIPHL_CAU-037070-0448	SRR29662386	New sequence	IV
IL-RIPHL_CAU-037070-0449	SRR29662385	New sequence	IV

IL-RIPHL_CAU-037070-0451	SRR29662383	New sequence	IV
IL-RIPHL_CAU-037070-0453	SRR29662381	New sequence	IV
IL-RIPHL_CAU-037070-0454	SRR29662380	New sequence	IV
IL-RIPHL_CAU-037070-0455	SRR29662379	New sequence	IV
IL-RIPHL_CAU-037070-0456	SRR29662378	New sequence	IV
IL-RIPHL_CAU-037070-0460	SRR29662374	New sequence	IV
IL-RIPHL_CAU-037070-0461	SRR29662373	New sequence	IV
IL-RIPHL_CAU-037070-0463	SRR29662370	New sequence	IV
IL-RIPHL_CAU-037070-0464	SRR29662369	New sequence	IV
IL-RIPHL_CAU-037070-0465	SRR29662368	New sequence	IV
IL-RIPHL_CAU-037070-0466	SRR29662367	New sequence	IV
IL-RIPHL_CAU-037070-0467	SRR29662366	New sequence	IV
IL-RIPHL_CAU-037070-0468	SRR29662365	New sequence	IV
IL-RIPHL_CAU-037070-0469	SRR29662364	New sequence	IV
IL-RIPHL_CAU-037070-0470	SRR29662363	New sequence	IV
IL-RIPHL_CAU-037070-0471	SRR29662362	New sequence	IV
IL-RIPHL_CAU-037070-0472	SRR29662361	New sequence	IV
IL-RIPHL_CAU-037070-0473	SRR29662359	New sequence	IV
IL-RIPHL_CAU-037070-0474	SRR29662358	New sequence	IV
IL-RIPHL_CAU-037070-0475	SRR29662357	New sequence	IV
IL-RIPHL_CAU-037070-0476	SRR29662356	New sequence	IV
IL-RIPHL_CAU-037070-0477	SRR29662355	New sequence	IV
IL-RIPHL_CAU-037070-0478	SRR29662354	New sequence	IV
IL-RIPHL_CAU-037070-0479	SRR29662353	New sequence	IV
IL-RIPHL_CAU-037070-0480	SRR29662352	New sequence	IV
IL-RIPHL_CAU-037070-0481	SRR29662351	New sequence	IV
IL-RIPHL_CAU-037070-0482	SRR29662350	New sequence	IV
IL-RIPHL_CAU-037070-0483	SRR29662348	New sequence	IV
IL-RIPHL_CAU-037070-0484	SRR29662347	New sequence	IV
IL-RIPHL_CAU-037070-0485	SRR29662346	New sequence	IV
IL-RIPHL_CAU-037070-0486	SRR29662345	New sequence	IV
IL-RIPHL_CAU-037070-0487	SRR29662344	New sequence	IV
IL-RIPHL_CAU-037070-0488	SRR29662343	New sequence	IV
IL-RIPHL_CAU-037070-0489	SRR29662342	New sequence	IV
IL-RIPHL_CAU-037070-0490	SRR29662341	New sequence	IV
IL-RIPHL_CAU-037070-0491	SRR29662340	New sequence	IV
IL-RIPHL_CAU-037070-0492	SRR29662339	New sequence	IV
IL-RIPHL_CAU-037070-0493	SRR29662571	New sequence	IV
IL-RIPHL_CAU-037070-0494	SRR29662570	New sequence	IV
IL-RIPHL_CAU-037070-0495	SRR29662569	New sequence	IV

IL-RIPHL_CAU-037070-0496	SRR29662568	New sequence	IV
IL-RIPHL_CAU-037070-0497	SRR29662567	New sequence	IV
IL-RIPHL_CAU-037070-0498	SRR29662566	New sequence	IV
IL-RIPHL_CAU-037070-0499	SRR29662565	New sequence	IV
IL-RIPHL_CAU-037070-0500	SRR29662564	New sequence	IV
IL-RIPHL_CAU-037070-0501	SRR29662563	New sequence	IV
IL-RIPHL_CAU-037070-0526	SRR29662503	New sequence	IV
IL-RIPHL_CAU-037070-0527	SRR29662502	New sequence	IV
IL-RIPHL_CAU-037070-0528	SRR29662501	New sequence	IV
IL-RIPHL_CAU-037070-0558	SRR29662470	New sequence	IV
IL-RIPHL_CAU-037070-0560	SRR29662468	New sequence	IV
IL-RIPHL_CAU-037070-0561	SRR29662467	New sequence	IV
IL-RIPHL_CAU-037070-0562	SRR29662466	New sequence	IV
IL-RIPHL_CAU-037070-0563	SRR29662465	New sequence	IV
IL-RIPHL_CAU-037070-0564	SRR29662464	New sequence	IV
IL-RIPHL_CAU-037070-0565	SRR29662462	New sequence	IV
IL-RIPHL_CAU-037070-0566	SRR29662461	New sequence	IV
IL-RIPHL_CAU-037070-0567	SRR29662460	New sequence	IV
IL-RIPHL_CAU-037070-0568	SRR29662459	New sequence	IV
IL-RIPHL_CAU-037070-0569	SRR29662458	New sequence	IV
IL-RIPHL_CAU-037070-0570	SRR29662457	New sequence	IV
IL-RIPHL_CAU-037070-0571	SRR29662456	New sequence	IV
IL-RIPHL_CAU-037070-0572	SRR29662455	New sequence	IV
IL-RIPHL_CAU-037070-0573	SRR29662454	New sequence	IV
IL-RIPHL_CAU-037070-0574	SRR29662453	New sequence	IV
IL-RIPHL_CAU-037070-0575	SRR29662451	New sequence	IV
IL-RIPHL_CAU-037070-0576	SRR29662450	New sequence	IV
IL-RIPHL_CAU-037070-0577	SRR29662449	New sequence	IV
IL-RIPHL_CAU-037070-0578	SRR29662448	New sequence	IV
IL-RIPHL_CAU-037070-0579	SRR29662447	New sequence	IV
IL-RIPHL_CAU-037070-0597	SRR29662426	New sequence	IV
IL-RIPHL_CAU-037070-0598	SRR29662425	New sequence	IV
IL-RIPHL_CAU-037070-0599	SRR29662424	New sequence	IV
IL-RIPHL_CAU-037070-0600	SRR29662423	New sequence	IV
IL-RIPHL_CAU-037070-0601	SRR29662422	New sequence	IV
IL-RIPHL_CAU-037070-0602	SRR29662421	New sequence	IV
IL-RIPHL_CAU-037070-0603	SRR29662420	New sequence	IV
IL-RIPHL_CAU-037070-0604	SRR29662419	New sequence	IV
IL-RIPHL_CAU-037070-0605	SRR29662417	New sequence	IV
IL-RIPHL_CAU-037070-0606	SRR29662416	New sequence	IV

IL-RIPHL_CAU-037070-0607	SRR29662415	New sequence	IV
IL-RIPHL_CAU-037070-0608	SRR29662542	New sequence	IV
IL-RIPHL_CAU-037070-0609	SRR29662541	New sequence	IV
IL-RIPHL_CAU-037070-0610	SRR29662540	New sequence	IV
IL-RIPHL_CAU-037070-0611	SRR29662539	New sequence	IV
IL-RIPHL_CAU-037070-0612	SRR29662538	New sequence	IV
IL-RIPHL_CAU-037070-0613	SRR29662537	New sequence	IV
IL-RIPHL_CAU-037070-0614	SRR29662536	New sequence	IV
IL-RIPHL_CAU-037070-0615	SRR29662534	New sequence	IV
IL-RIPHL_CAU-037070-0616	SRR29662533	New sequence	IV
IL-RIPHL_CAU-037070-0617	SRR29662532	New sequence	IV
IL-RIPHL_CAU-037070-0618	SRR29662531	New sequence	IV
IL-RIPHL_CAU-037070-0619	SRR29662530	New sequence	IV
IL-RIPHL_CAU-037070-0620	SRR29662529	New sequence	IV
IL-RIPHL_CAU-037070-0621	SRR29662528	New sequence	IV
IL-RIPHL_CAU-037070-0622	SRR29662527	New sequence	IV
IL-RIPHL_CAU-037070-0623	SRR29662526	New sequence	IV
IL-RIPHL_CAU-037070-0624	SRR29662525	New sequence	IV
IL-RIPHL_CAU-037070-0625	SRR29662523	New sequence	IV
IL-RIPHL_CAU-038070-0580	SRR29662446	New sequence	IV
IL-RIPHL_CAU-038070-0581	SRR29662445	New sequence	IV
IL-RIPHL_CAU-038070-0582	SRR29662444	New sequence	IV
IL-RIPHL_CAU-038070-0584	SRR29662442	New sequence	IV
IL-RIPHL_CAU-038070-0585	SRR29662440	New sequence	IV
IL-RIPHL_CAU-038070-0586	SRR29662439	New sequence	IV
IL-RIPHL_CAU-038070-0587	SRR29662438	New sequence	IV
IL-RIPHL_CAU-038070-0588	SRR29662437	New sequence	IV
IL-RIPHL_CAU-038070-0589	SRR29662436	New sequence	IV
IL-RIPHL_CAU-038070-0590	SRR29662435	New sequence	IV
IL-RIPHL_CAU-038070-0592	SRR29662433	New sequence	IV
IL-RIPHL_CAU-038070-0594	SRR29662431	New sequence	IV
IL-RIPHL_CAU-038070-0595	SRR29662428	New sequence	IV
IL-RIPHL_CAU-038070-0596	SRR29662427	New sequence	IV
IL-RIPHL_CAU-039070-0393	SRR29662511	New sequence	IV
IL-RIPHL_CAU-039070-0394	SRR29662404	New sequence	IV
IL-RIPHL_CAU-039070-0395	SRR29662393	New sequence	IV
IL-RIPHL_CAU-039070-0396	SRR29662382	New sequence	IV
IL-RIPHL_CAU-039070-0512	SRR29662551	New sequence	IV
IL-RIPHL_CAU-039070-0513	SRR29662549	New sequence	IV
IL-RIPHL_CAU-039070-0514	SRR29662548	New sequence	IV

IL-RIPHL_CAU-039070-0515	SRR29662547	New sequence	IV
IL-RIPHL_CAU-039070-0516	SRR29662546	New sequence	IV
IL-RIPHL_CAU-039070-0517	SRR29662545	New sequence	IV
IL-RIPHL_CAU-039070-0518	SRR29662544	New sequence	IV
IL-RIPHL_CAU-039070-0519	SRR29662543	New sequence	IV
IL-RIPHL_CAU-039070-0520	SRR29662510	New sequence	IV
IL-RIPHL_CAU-039070-0521	SRR29662509	New sequence	IV
IL-RIPHL_CAU-039070-0522	SRR29662508	New sequence	IV
IL-RIPHL_CAU-039070-0523	SRR29662506	New sequence	IV
IL-RIPHL_CAU-039070-0524	SRR29662505	New sequence	IV
IL-RIPHL_CAU-039070-0525	SRR29662504	New sequence	IV
IL-RIPHL-CAU-012012-0386	N/A	Illinois contextual	
IL-RIPHL-CAU-012012-0387	N/A	Illinois contextual	
IL-RIPHL-CAU-012012-0388	N/A	Illinois contextual	
IL-RIPHL-CAU-012012-0389	N/A	Illinois contextual	
IL-RIPHL-CAU-120002	SRR22393499	Illinois contextual	
IL-RIPHL-CAU-120003	SRR22393498	Illinois contextual	
IL-RIPHL-CAU-120013	SRR24673390	Illinois contextual	
IL-RIPHL-CAU-120015	SRR24673377	Illinois contextual	
IL-RIPHL-CAU-120019	SRR24673432	Illinois contextual	
IL-RIPHL-CAU-120021	SRR24673430	Illinois contextual	
IL-RIPHL-CAU-120025	SRR24673426	Illinois contextual	
IL-RIPHL-CAU-120032	SRR24673418	Illinois contextual	
IL-RIPHL-CAU-120033	SRR24673417	Illinois contextual	
IL-RIPHL-CAU-120050	SRR24673398	Illinois contextual	
IL-RIPHL-CAU-120062	SRR24673386	Illinois contextual	
IL-RIPHL-CAU-70-0070	SRR27223802	New sequence	IV
IL-RIPHL-CAU-70-0071	SRR27223801	New sequence	IV
IL-RIPHL-CAU-70-0072	SRR27223572	New sequence	IV
IL-RIPHL-CAU-70-0073	SRR27223611	New sequence	IV
IL-RIPHL-CAU-70-0074	SRR27223600	New sequence	IV
IL-RIPHL-CAU-70-0075	SRR27223589	New sequence	IV
IL-RIPHL-CAU-70-0076	SRR27223546	New sequence	IV
IL-RIPHL-CAU-70-0077	SRR27223535	New sequence	IV
IL-RIPHL-CAU-70-0078	SRR27223738	New sequence	IV
IL-RIPHL-CAU-70-0079	SRR27223727	New sequence	IV
IL-RIPHL-CAU-70-0080	SRR27223662	New sequence	IV
IL-RIPHL-CAU-70-0081	SRR27223800	New sequence	IV
IL-RIPHL-CAU-70-0082	SRR27223789	New sequence	IV
IL-RIPHL-CAU-70-0083	SRR27223778	New sequence	IV

IL-RIPHL-CAU-70-0084	SRR27223713	New sequence	IV
IL-RIPHL-CAU-70-0085	SRR27223702	New sequence	IV
IL-RIPHL-CAU-70-0086	SRR27223691	New sequence	IV
IL-RIPHL-CAU-70-0087	SRR27223648	New sequence	IV
IL-RIPHL-CAU-70-0088	SRR27223637	New sequence	IV
IL-RIPHL-CAU-70-0089	SRR27223626	New sequence	IV
IL-RIPHL-CAU-70-0090	SRR27223583	New sequence	IV
IL-RIPHL-CAU-70-0091	SRR27223571	New sequence	IV
IL-RIPHL-CAU-70-0092	SRR27223560	New sequence	IV
IL-RIPHL-CAU-70-0093	SRR27223763	New sequence	IV
IL-RIPHL-CAU-70-0094	SRR27223752	New sequence	IV
IL-RIPHL-CAU-70-0095	SRR27223741	New sequence	IV
IL-RIPHL-CAU-70-0096	SRR27223676	New sequence	IV
IL-RIPHL-CAU-70-0097	SRR27223665	New sequence	IV
IL-RIPHL-CAU-70-0098	SRR27223654	New sequence	IV
IL-RIPHL-CAU-70-0099	SRR27223661	New sequence	IV
IL-RIPHL-CAU-70-0100	SRR27223613	New sequence	IV
IL-RIPHL-CAU-70-0101	SRR27223612	New sequence	IV
IL-RIPHL-CAU-70-0102	SRR27223610	New sequence	IV
IL-RIPHL-CAU-70-0103	SRR27223609	New sequence	IV
IL-RIPHL-CAU-70-0104	SRR27223608	New sequence	IV
IL-RIPHL-CAU-70-0105	SRR27223607	New sequence	IV
IL-RIPHL-CAU-70-0106	SRR27223606	New sequence	IV
IL-RIPHL-CAU-70-0107	SRR27223605	New sequence	IV
IL-RIPHL-CAU-70-0108	SRR27223604	New sequence	IV
IL-RIPHL-CAU-70-0109	SRR27223603	New sequence	IV
IL-RIPHL-CAU-70-0110	SRR27223602	New sequence	IV
IL-RIPHL-CAU-70-0111	SRR27223601	New sequence	IV
IL-RIPHL-CAU-70-0112	SRR27223599	New sequence	IV
IL-RIPHL-CAU-70-0113	SRR27223598	New sequence	IV
IL-RIPHL-CAU-70-0114	SRR27223597	New sequence	IV
IL-RIPHL-CAU-70-0115	SRR27223596	New sequence	IV
IL-RIPHL-CAU-70-0116	SRR27223595	New sequence	IV
IL-RIPHL-CAU-70-0117	SRR27223594	New sequence	IV
IL-RIPHL-CAU-70-0118	SRR27223593	New sequence	IV
IL-RIPHL-CAU-70-0119	SRR27223592	New sequence	IV
IL-RIPHL-CAU-70-0120	SRR27223591	New sequence	IV
IL-RIPHL-CAU-70-0121	SRR27223590	New sequence	IV
IL-RIPHL-CAU-70-0122	SRR27223556	New sequence	IV
IL-RIPHL-CAU-70-0123	SRR27223555	New sequence	IV

IL-RIPHL-CAU-70-0124	SRR27223554	New sequence	IV
IL-RIPHL-CAU-70-0125	SRR27223553	New sequence	IV
IL-RIPHL-CAU-70-0126	SRR27223552	New sequence	IV
IL-RIPHL-CAU-70-0127	SRR27223551	New sequence	IV
IL-RIPHL-CAU-70-0128	SRR27223550	New sequence	IV
IL-RIPHL-CAU-70-0129	SRR27223549	New sequence	IV
IL-RIPHL-CAU-70-0130	SRR27223548	New sequence	IV
IL-RIPHL-CAU-70-0131	SRR27223547	New sequence	IV
IL-RIPHL-CAU-70-0132	SRR27223545	New sequence	IV
IL-RIPHL-CAU-70-0134	SRR27223544	New sequence	IV
IL-RIPHL-CAU-70-0135	SRR27223543	New sequence	IV
IL-RIPHL-CAU-70-0136	SRR27223542	New sequence	IV
IL-RIPHL-CAU-70-0137	SRR27223541	New sequence	IV
IL-RIPHL-CAU-70-0138	SRR27223540	New sequence	IV
IL-RIPHL-CAU-70-0139	SRR27223539	New sequence	IV
IL-RIPHL-CAU-70-0140	SRR27223538	New sequence	IV
IL-RIPHL-CAU-70-0141	SRR27223537	New sequence	IV
IL-RIPHL-CAU-70-0142	SRR27223536	New sequence	IV
IL-RIPHL-CAU-70-0143	SRR27223534	New sequence	IV
IL-RIPHL-CAU-70-0144	SRR27223533	New sequence	IV
IL-RIPHL-CAU-70-0145	SRR27223532	New sequence	IV
IL-RIPHL-CAU-70-0146	SRR27223531	New sequence	IV
IL-RIPHL-CAU-70-0147	SRR27223530	New sequence	IV
IL-RIPHL-CAU-70-0148	SRR27223529	New sequence	IV
IL-RIPHL-CAU-70-0149	SRR27223528	New sequence	IV
IL-RIPHL-CAU-70-0150	SRR27223527	New sequence	IV
IL-RIPHL-CAU-70-0151	SRR27223526	New sequence	IV
IL-RIPHL-CAU-70-0152	SRR27223525	New sequence	IV
IL-RIPHL-CAU-70-0153	SRR27223737	New sequence	IV
IL-RIPHL-CAU-70-0154	SRR27223736	New sequence	IV
IL-RIPHL-CAU-70-0155	SRR27223660	New sequence	IV
IL-RIPHL-CAU-70-0156	SRR27223735	New sequence	IV
IL-RIPHL-CAU-70-0157	SRR27223734	New sequence	IV
IL-RIPHL-CAU-70-0158	SRR27223733	New sequence	IV
IL-RIPHL-CAU-70-0159	SRR27223732	New sequence	IV
IL-RIPHL-CAU-70-0160	SRR27223731	New sequence	IV
IL-RIPHL-CAU-70-0161	SRR27223730	New sequence	IV
IL-RIPHL-CAU-70-0162	SRR27223729	New sequence	IV
IL-RIPHL-CAU-70-0163	SRR27223728	New sequence	IV
IL-RIPHL-CAU-70-0164	SRR27223726	New sequence	IV

IL-RIPHL-CAU-70-0166	SRR27223725	New sequence	IV
IL-RIPHL-CAU-70-0167	SRR27223724	New sequence	IV
IL-RIPHL-CAU-70-0168	SRR27223723	New sequence	IV
IL-RIPHL-CAU-70-0169	SRR27223722	New sequence	IV
IL-RIPHL-CAU-70-0170	SRR27223721	New sequence	IV
IL-RIPHL-CAU-70-0171	SRR27223720	New sequence	IV
IL-RIPHL-CAU-70-0172	SRR27223719	New sequence	IV
IL-RIPHL-CAU-70-0173	SRR27223718	New sequence	IV
IL-RIPHL-CAU-70-0174	SRR27223717	New sequence	IV
IL-RIPHL-CAU-70-0175	SRR27223799	New sequence	IV
IL-RIPHL-CAU-70-0176	SRR27223798	New sequence	IV
IL-RIPHL-CAU-70-0177	SRR27223797	New sequence	IV
IL-RIPHL-CAU-70-0178	SRR27223796	New sequence	IV
IL-RIPHL-CAU-70-0179	SRR27223795	New sequence	IV
IL-RIPHL-CAU-70-0180	SRR27223794	New sequence	IV
IL-RIPHL-CAU-70-0181	SRR27223793	New sequence	IV
IL-RIPHL-CAU-70-0182	SRR27223792	New sequence	IV
IL-RIPHL-CAU-70-0183	SRR27223791	New sequence	IV
IL-RIPHL-CAU-70-0184	SRR27223790	New sequence	IV
IL-RIPHL-CAU-70-0185	SRR27223788	New sequence	IV
IL-RIPHL-CAU-70-0186	SRR27223787	New sequence	IV
IL-RIPHL-CAU-70-0187	SRR27223786	New sequence	IV
IL-RIPHL-CAU-70-0188	SRR27223785	New sequence	IV
IL-RIPHL-CAU-70-0189	SRR27223784	New sequence	IV
IL-RIPHL-CAU-70-0190	SRR27223783	New sequence	IV
IL-RIPHL-CAU-70-0191	SRR27223782	New sequence	IV
IL-RIPHL-CAU-70-0192	SRR27223781	New sequence	IV
IL-RIPHL-CAU-70-0193	SRR27223780	New sequence	IV
IL-RIPHL-CAU-70-0194	SRR27223779	New sequence	IV
IL-RIPHL-CAU-70-0195	SRR27223777	New sequence	IV
IL-RIPHL-CAU-70-0196	SRR27223776	New sequence	IV
IL-RIPHL-CAU-70-0197	SRR27223775	New sequence	IV
IL-RIPHL-CAU-70-0198	SRR27223774	New sequence	IV
IL-RIPHL-CAU-70-0199	SRR27223773	New sequence	IV
IL-RIPHL-CAU-70-0200	SRR27223772	New sequence	IV
IL-RIPHL-CAU-70-0201	SRR27223771	New sequence	IV
IL-RIPHL-CAU-70-0202	SRR27223716	New sequence	IV
IL-RIPHL-CAU-70-0203	SRR27223715	New sequence	IV
IL-RIPHL-CAU-70-0204	SRR27223714	New sequence	IV
IL-RIPHL-CAU-70-0205	SRR27223712	New sequence	IV

IL-RIPHL-CAU-70-0206	SRR27223711	New sequence	IV
IL-RIPHL-CAU-70-0207	SRR27223710	New sequence	IV
IL-RIPHL-CAU-70-0208	SRR27223659	New sequence	IV
IL-RIPHL-CAU-70-0209	SRR27223709	New sequence	IV
IL-RIPHL-CAU-70-0210	SRR27223708	New sequence	IV
IL-RIPHL-CAU-70-0211	SRR27223707	New sequence	IV
IL-RIPHL-CAU-70-0212	SRR27223706	New sequence	IV
IL-RIPHL-CAU-70-0213	SRR27223705	New sequence	IV
IL-RIPHL-CAU-70-0214	SRR27223704	New sequence	IV
IL-RIPHL-CAU-70-0215	SRR27223703	New sequence	IV
IL-RIPHL-CAU-70-0216	SRR27223701	New sequence	IV
IL-RIPHL-CAU-70-0217	SRR27223700	New sequence	IV
IL-RIPHL-CAU-70-0218	SRR27223699	New sequence	IV
IL-RIPHL-CAU-70-0219	SRR27223698	New sequence	IV
IL-RIPHL-CAU-70-0220	SRR27223697	New sequence	IV
IL-RIPHL-CAU-70-0221	SRR27223696	New sequence	IV
IL-RIPHL-CAU-70-0222	SRR27223695	New sequence	IV
IL-RIPHL-CAU-70-0223	SRR27223694	New sequence	IV
IL-RIPHL-CAU-70-0224	SRR27223693	New sequence	IV
IL-RIPHL-CAU-70-0225	SRR27223692	New sequence	IV
IL-RIPHL-CAU-70-0226	SRR27223690	New sequence	IV
IL-RIPHL-CAU-70-0227	SRR27223689	New sequence	IV
IL-RIPHL-CAU-70-0228	SRR27223688	New sequence	IV
IL-RIPHL-CAU-70-0229	SRR27223687	New sequence	IV
IL-RIPHL-CAU-70-0230	SRR27223686	New sequence	IV
IL-RIPHL-CAU-70-0231	SRR27223685	New sequence	IV
IL-RIPHL-CAU-70-0232	SRR27223652	New sequence	IV
IL-RIPHL-CAU-70-0233	SRR27223651	New sequence	IV
IL-RIPHL-CAU-70-0234	SRR27223650	New sequence	IV
IL-RIPHL-CAU-70-0235	SRR27223649	New sequence	IV
IL-RIPHL-CAU-70-0236	SRR27223647	New sequence	IV
IL-RIPHL-CAU-70-0237	SRR27223646	New sequence	IV
IL-RIPHL-CAU-70-0238	SRR27223645	New sequence	IV
IL-RIPHL-CAU-70-0239	SRR27223644	New sequence	IV
IL-RIPHL-CAU-70-0240	SRR27223643	New sequence	IV
IL-RIPHL-CAU-70-0241	SRR27223642	New sequence	IV
IL-RIPHL-CAU-70-0242	SRR27223641	New sequence	IV
IL-RIPHL-CAU-70-0243	SRR27223640	New sequence	IV
IL-RIPHL-CAU-70-0244	SRR27223639	New sequence	IV
IL-RIPHL-CAU-70-0245	SRR27223638	New sequence	IV

IL-RIPHL-CAU-70-0246	SRR27223636	New sequence	IV
IL-RIPHL-CAU-70-0247	SRR27223635	New sequence	IV
IL-RIPHL-CAU-70-0248	SRR27223634	New sequence	IV
IL-RIPHL-CAU-70-0249	SRR27223633	New sequence	IV
IL-RIPHL-CAU-70-0250	SRR27223632	New sequence	IV
IL-RIPHL-CAU-70-0251	SRR27223631	New sequence	IV
IL-RIPHL-CAU-70-0252	SRR27223630	New sequence	IV
IL-RIPHL-CAU-70-0253	SRR27223629	New sequence	IV
IL-RIPHL-CAU-70-0254	SRR27223628	New sequence	IV
IL-RIPHL-CAU-70-0255	SRR27223627	New sequence	IV
IL-RIPHL-CAU-70-0256	SRR27223625	New sequence	IV
IL-RIPHL-CAU-70-0257	SRR27223624	New sequence	IV
IL-RIPHL-CAU-70-0258	SRR27223623	New sequence	IV
IL-RIPHL-CAU-70-0259	SRR27223622	New sequence	IV
IL-RIPHL-CAU-70-0260	SRR27223621	New sequence	IV
IL-RIPHL-CAU-70-0261	SRR27223588	New sequence	IV
IL-RIPHL-CAU-70-0262	SRR27223587	New sequence	IV
IL-RIPHL-CAU-70-0263	SRR27223586	New sequence	IV
IL-RIPHL-CAU-70-0264	SRR27223585	New sequence	IV
IL-RIPHL-CAU-70-0265	SRR27223584	New sequence	IV
IL-RIPHL-CAU-70-0266	SRR27223582	New sequence	IV
IL-RIPHL-CAU-70-0267	SRR27223581	New sequence	IV
IL-RIPHL-CAU-70-0268	SRR27223580	New sequence	IV
IL-RIPHL-CAU-70-0269	SRR27223579	New sequence	IV
IL-RIPHL-CAU-70-0270	SRR27223578	New sequence	IV
IL-RIPHL-CAU-70-0271	SRR27223577	New sequence	IV
IL-RIPHL-CAU-70-0272	SRR27223576	New sequence	IV
IL-RIPHL-CAU-70-0273	SRR27223575	New sequence	IV
IL-RIPHL-CAU-70-0274	SRR27223574	New sequence	IV
IL-RIPHL-CAU-70-0275	SRR27223573	New sequence	IV
IL-RIPHL-CAU-70-0276	SRR27223570	New sequence	IV
IL-RIPHL-CAU-70-0277	SRR27223569	New sequence	IV
IL-RIPHL-CAU-70-0278	SRR27223568	New sequence	IV
IL-RIPHL-CAU-70-0279	SRR27223567	New sequence	IV
IL-RIPHL-CAU-70-0280	SRR27223566	New sequence	IV
IL-RIPHL-CAU-70-0281	SRR27223565	New sequence	IV
IL-RIPHL-CAU-70-0282	SRR27223564	New sequence	IV
IL-RIPHL-CAU-70-0283	SRR27223563	New sequence	IV
IL-RIPHL-CAU-70-0284	SRR27223562	New sequence	IV
IL-RIPHL-CAU-70-0285	SRR27223561	New sequence	IV

IL-RIPHL-CAU-70-0286	SRR27223559	New sequence	IV
IL-RIPHL-CAU-70-0287	SRR27223558	New sequence	IV
IL-RIPHL-CAU-70-0288	SRR27223557	New sequence	IV
IL-RIPHL-CAU-70-0289	SRR27223770	New sequence	IV
IL-RIPHL-CAU-70-0290	SRR27223769	New sequence	IV
IL-RIPHL-CAU-70-0291	SRR27223768	New sequence	IV
IL-RIPHL-CAU-70-0292	SRR27223767	New sequence	IV
IL-RIPHL-CAU-70-0293	SRR27223766	New sequence	IV
IL-RIPHL-CAU-70-0294	SRR27223765	New sequence	IV
IL-RIPHL-CAU-70-0295	SRR27223764	New sequence	IV
IL-RIPHL-CAU-70-0296	SRR27223762	New sequence	IV
IL-RIPHL-CAU-70-0297	SRR27223761	New sequence	IV
IL-RIPHL-CAU-70-0298	SRR27223760	New sequence	IV
IL-RIPHL-CAU-70-0299	SRR27223759	New sequence	IV
IL-RIPHL-CAU-70-0300	SRR27223758	New sequence	IV
IL-RIPHL-CAU-70-0301	SRR27223757	New sequence	IV
IL-RIPHL-CAU-70-0302	SRR27223756	New sequence	IV
IL-RIPHL-CAU-70-0303	SRR27223755	New sequence	IV
IL-RIPHL-CAU-70-0305	SRR27223754	New sequence	IV
IL-RIPHL-CAU-70-0305	SRR27223754	New sequence	IV
IL-RIPHL-CAU-70-0306	SRR27223753	New sequence	IV
IL-RIPHL-CAU-70-0307	SRR27223751	New sequence	IV
IL-RIPHL-CAU-70-0308	SRR27223750	New sequence	IV
IL-RIPHL-CAU-70-0309	SRR27223749	New sequence	IV
IL-RIPHL-CAU-70-0310	SRR27223748	New sequence	IV
IL-RIPHL-CAU-70-0311	SRR27223657	New sequence	IV
IL-RIPHL-CAU-70-0312	SRR27223747	New sequence	IV
IL-RIPHL-CAU-70-0314	SRR27223746	New sequence	IV
IL-RIPHL-CAU-70-0315	SRR27223745	New sequence	IV
IL-RIPHL-CAU-70-0316	SRR27223744	New sequence	IV
IL-RIPHL-CAU-70-0317	SRR27223743	New sequence	IV
IL-RIPHL-CAU-70-0318	SRR27223742	New sequence	IV
IL-RIPHL-CAU-70-0319	SRR27223740	New sequence	IV
IL-RIPHL-CAU-70-0320	SRR27223656	New sequence	IV
IL-RIPHL-CAU-70-0321	SRR27223655	New sequence	IV
IL-RIPHL-CAU-70-0322	SRR27223739	New sequence	IV
IL-RIPHL-CAU-70-0323	SRR27223684	New sequence	IV
IL-RIPHL-CAU-70-0324	SRR27223683	New sequence	IV
IL-RIPHL-CAU-70-0325	SRR27223682	New sequence	IV
IL-RIPHL-CAU-70-0326	SRR27223681	New sequence	IV

IL-RIPHL-CAU-70-0327	SRR27223653	New sequence	IV
IL-RIPHL-CAU-70-0328	SRR27223680	New sequence	IV
IL-RIPHL-CAU-70-0329	SRR27223679	New sequence	IV
IL-RIPHL-CAU-70-0330	SRR27223678	New sequence	IV
IL-RIPHL-CAU-70-0331	SRR27223677	New sequence	IV
IL-RIPHL-CAU-70-0332	SRR27223675	New sequence	IV
IL-RIPHL-CAU-70-0333	SRR27223674	New sequence	IV
IL-RIPHL-CAU-70-0334	SRR27223673	New sequence	IV
IL-RIPHL-CAU-70-0335	SRR27223672	New sequence	IV
IL-RIPHL-CAU-038070-0583	SRR29662443	New sequence	III
IL-RIPHL-CAU-038070-0591	SRR29662434	New sequence	III
IL-RIPHL-CAU-038070-0593	SRR29662432	New sequence	III
